# Causal Relationships between Polyunsaturated Fatty Acids and Colon Polyps: A Two-Sample Mendelian Randomization Study

**DOI:** 10.3390/nu16132033

**Published:** 2024-06-27

**Authors:** Na Shen, Qinwen Ba, Yanjun Lu

**Affiliations:** Department of Laboratory Medicine, Tongji Hospital, Tongji Medical College, Huazhong University of Science and Technology, Wuhan 430030, China; shenna@tjh.tjmu.edu.cn (N.S.); bqw@hust.edu.cn (Q.B.)

**Keywords:** colon polyps, mendelian randomization, fatty acids, causality

## Abstract

Background: Epidemiological studies have shown that fatty acids, especially polyunsaturated fatty acids (PUFAs), influence colorectal carcinogenesis. Colon polyps, particularly those identified as precancerous, are a frequently encountered phenomenon associated with PUFAs. However, the results are inconsistent. Therefore, we investigated the effect of PUFAs on colon polyps in individuals of European ancestry. Methods: Single nucleotide polymorphisms correlating with PUFAs and colon polyps were derived from extensive genome-wide association studies, encompassing a discovery cohort of 135,006 samples and a corresponding validation set with 114,999 samples. Causality was assessed by employing a range of techniques, such as inverse variance weighted (IVW), weighted median, MR-Egger, and simple and weighted modes. The analysis was complemented with sensitivity checks using leave-one-out and heterogeneity evaluation through MR-IVW and Cochran’s Q. Results: A thorough analysis was performed to examine the causal effects of PUFAs on the development of colon polyps. The findings indicated that levels of Omega-3 fatty acids (OR = 1.0014, 95% CI 1.0004–1.0024, *p* = 0.004), the ratio of Docosahexaenoic acid (DHA)/total fatty acids (FAs) (DHA/totalFA; OR = 1.0015, 95% CI 1.0002–1.0028, *p* = 0.023), and the ratio of Omega-3/totalFA (Omega-3/totalFA; OR = 1.0013, 95% CI 1.0003–1.0022, *p* = 0.010) were identified as biomarkers associated with an increased risk of colon polyps. Conversely, the ratio of Omega-6/Omega-3 (OR = 0.9986, 95% CI 0.9976–0.9995, *p* = 0.003) and the ratio of Linoleic acid (LA)/totalFA (LA/totalFA; OR = 0.9981, 95% CI 0.9962–0.9999, *p* = 0.044) were negatively associated with susceptibility to colon polyps. The MR-Egger and MR-IVW analysis revealed that pleiotropy and heterogeneity did not significantly impact the outcomes. Conclusion: This study has uncovered a possible adverse effect of PUFAs, notably Omega-3, on the formation of colon polyps. Elevated Omega-3 levels were correlated with a heightened risk of colon polyps.

## 1. Introduction

A primary objective of colorectal cancer (CRC) screening is the prevention of CRC through the detection and removal of precancerous colon polyps, which are identified in approximately 50% of screening examinations [1]. Colon polyps, identified as abnormal growths on the mucosal lining of the colon or rectum, are a common occurrence, particularly prevalent in those above the age of 50. These growths are frequently discovered during standard screening protocols, including procedures such as colonoscopy [2].

Colon polyps are classified into two main types: neoplastic and non-neoplastic, distinguished by their morphological characteristics and the associated risk of malignant transformation. Inflammatory, hamartomatous, lymphoid, mucosal prolapse, and hyperplastic polyps predominantly make up the non-neoplastic category. Conversely, neoplastic polyps, characterized by their potential for malignant transformation, are primarily of the adenomatous and serrated types [3].

Epidemiological studies have identified several risk factors for colon polyps, including advancing age, a positive family history, and a personal history of such lesions. Individuals with a history of colon polyps or inflammatory bowel disease are at a higher risk [4]. Additionally, lifestyle factors contribute to this risk, particularly a diet rich in red or processed meats and low in fiber, as well as behaviors such as obesity, smoking, and excessive alcohol consumption [5]. Furthermore, certain genetic syndromes, including familial adenomatous polyposis (FAP) and Lynch syndrome, are known to predispose individuals to the development of colon polyps [6].

Polyunsaturated fatty acids (PUFAs), a subset of fatty acids, are believed to affect the development of colorectal cancer by altering various factors, which encompass cell signaling, inflammation, and the gut microbiota [7]. Previous studies indicate that increased dietary intake of n-6 PUFAs may raise the risk of developing conventional adenomas [8]. Conversely, n-3 PUFAs have been implicated in offering protection against such adenomas [9]. Nevertheless, other studies have yielded inconsistent findings [10,11]. Compared to self-reported dietary intake, blood tests provide a more accurate reflection of an individual’s internal exposure to PUFAs. A recent cross-sectional analysis discovered a dose-dependent link between plasma PUFA levels and the incidence of colonic adenomas in a cohort of 126 men who underwent screening colonoscopy [12]. Several prospective studies have evaluated the association between serum/plasma biomarkers of PUFAs and the risk of colorectal neoplasia, yet the findings have been inconsistent [13,14]. These conflicting study outcomes imply that dietary fatty acid composition may be a complex factor in determining cancer risk.

Mendelian randomization (MR) is an epidemiological and genetic research technique employed to evaluate causality between an exposure and an outcome [15]. This method employs genetic variants to serve as instrumental variables, thereby examining the influence of alterable risk factors on health outcomes in observational research. MR capitalizes on genetic variations that are stochastically allocated during meiosis, thereby potentially circumventing common challenges, such as confounding and reverse causation, encountered in traditional observational research [16]. The majority of CRCs are thought to arise through the benign precursor, the colorectal polyp—encompassing conventional adenomas and serrated polyps—in a process that may span approximately 10 years [17]. Therefore, it is hypothesized that PUFAs may also play a role in the development of colorectal polyps, presenting a potential early intervention point to ameliorate the initial stages of colorectal carcinogenesis. In this research, we performed a two-sample MR analysis, leveraging two separate genome-wide association study (GWAS) datasets, to reveal the potential causal association between different fatty acid levels, with a particular emphasis on PUFAs, and the occurrence of colon polyps.

## 2. Methods

### 2.1. Study Design

**Figure 1:**
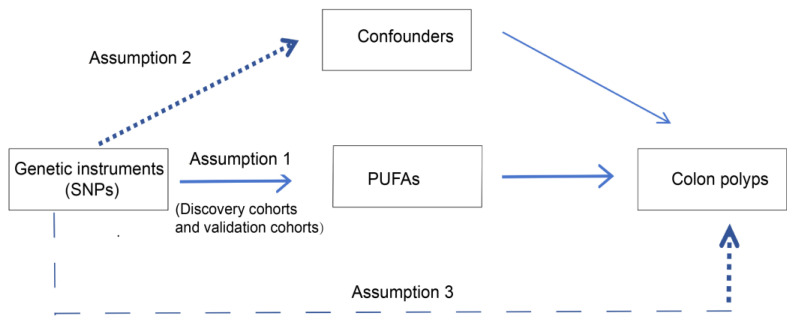
Flowchart of the Mendelian randomization (MR) study. SNPs: single nucleotide polymorphisms, PUFAs: polyunsaturated fatty acids.

To investigate the potential causal associations between PUFAs and the risk of colon polyps, we conducted a two-sample MR study. This approach is predicated on three fundamental assumptions: genetic variation should be strongly associated with the exposure of interest, genetic variants should not act as confounders, and the relationship between genetic variants and outcomes should be mediated solely via the exposure. For our analysis, we utilized two distinct exposure datasets, namely the discovery and validation cohorts. Figure 1 illustrates the overall study design.

### 2.2. Data Sources and Participants

GWAS summary data pertaining to the exposure factors, including monounsaturated fatty acid (MUFA), Omega-3 fatty acid, Omega-6 fatty acid, PUFA, and various ratios including Docosahexaenoic acid (DHA)/total fatty acid (FA), Linoleic acid (LA)/totalFA, monounsaturated fatty acid (MUFA)/totalFA, Omega-3/totalFA, Omega-6/Omega-3, Omega-6/totalFA, PUFA/MUFA, PUFA/totalFA, saturated fatty acid (SFA)/totalFA, levels of SFA, and total fatty acid were sourced from Open GWAS (https://gwas.mrcieu.ac.uk/) on 19 April 2024. Our analysis encompassed the discovery exposure dataset, containing 115,006 samples and 11,590,339 single nucleotide polymorphisms (SNPs), and the validation exposure dataset, containing 114,999 samples and 12,321,875 SNPs. The GWAS data, which specifically targeted “Diagnosis-Main ICD10: K63.5 Polyp of colon”, included 463,010 cases and 9,851,867 SNPs associated with the outcome of colon polyps. The participants were exclusively of European descent, reducing the likelihood of ethnic bias. The use of separate databases suggests no overlap in the study populations. All datasets were sourced from the IEU OpenGWAS platform. Detailed descriptions of the discovery exposure and outcome datasets are delineated in Table 1 and the validation outcome datasets are shown in Table S1. All studies were approved by the relevant institutional review boards, and written informed consent was obtained from all participants.

### 2.3. Selection of SNPs

To ensure robust instrumental variables (IVs) for PUFAs and statistical power, only SNPs with significant associations (*p* < 5 × 10^−8^) were considered IVs. Subsequently, a stringent linkage disequilibrium (LD) clumping threshold, which was used to evaluate the non-random association of alleles at different loci, was applied for the selection of genetic instruments, specifically an r^2^ threshold of less than 0.01 within a window size of 10 Mb. This was accomplished using the ‘clump_data’ function within the TwoSampleMR R package.

We then harmonized the exposure and outcome datasets to ascertain the effects of genetic instruments on colon polyps, simultaneously removing palindromic SNPs, which are defined by alleles that correspond to mirrored nucleotide sequences in the DNA molecule. In MR analysis, the exclusion of palindromic SNPs is deemed necessary to ensure robust results. When SNPs predictive of specific PUFAs in the outcome GWAS were eliminated, proxy SNPs in high LD (LD, r^2^ > 0.8) were utilized. The strength of the instruments for exposures was assessed using the proportion of variance explained by the SNPs (R^2^) and the F statistic. Typically, SNPs that have an F statistic above 10 are deemed sufficiently strong instruments for MR analysis.

### 2.4. MR Analysis

In the MR analysis, we employed five unique MR methods to examine the causal impact of PUFAs on colon polyps. The primary technique utilized was the inverse variance weighted (IVW) method, which is conventional for summary statistics data and allows for the calculation of causal effect sizes without necessitating individual-level data. The MR-Egger method was utilized, which offers valid causal estimates even with pleiotropy, especially when the intercept’s *p*-value is below 0.05, as it identifies and corrects potential horizontal pleiotropy. Additionally, we employed the weighted median method, which strengthens causal estimates in MR analyses, even if up to fifty percent of the instrumental variables are potentially invalid. The simple mode and weighted mode methods were included, assuming that the largest cluster of similar individual instrument causal effect estimates originates from valid instruments, serving as supplementary tools in our analysis. The MR analysis results were reported with *p*-values, odds ratios (ORs), and 95% confidence intervals (CIs). Heterogeneity was evaluated using Cochran’s Q statistic in both the MR-Egger and IVW methods, where a *p*-value exceeding 0.05 indicated no significant heterogeneity. The closer the intercept value is to zero, the less influence genetic pleiotropy exerts. The leave-one-out analysis verified that the causal relationship between PUFAs and colon polyps was not skewed by any individual SNP. Notable SNPs with significant impact were marked for detailed reassessment. We used the MR platform to create forest and scatter plots demonstrating the relationship between SNPs, PUFAs, and colon polyp risk.

### 2.5. Statistical Analysis

Statistical analyses were conducted using the TwoSampleMR package (v0.4.25) in R (v3.6.2).

## 3. Result

### 3.1. Causal Effects of Fatty Acids on Colon Polyps

**Figure 2:**
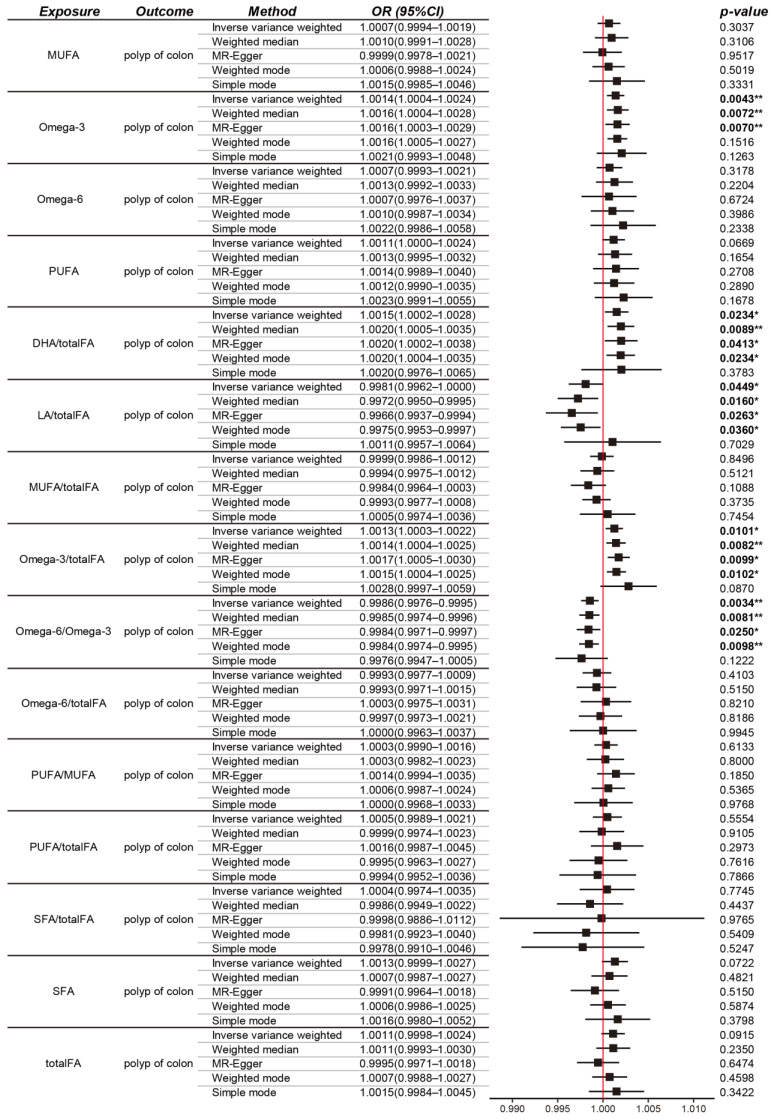
Mendelian randomization (MR) analysis showing the causal relationship between different types of FAs and colon polyp outcomes. MUFA: monounsaturated fatty acid; PUFA: polyunsaturated fatty acid; DHA: Docosahexaenoic acid; LA: Linoleic acid; FA: fatty acid; SFA: saturated fatty acid; OR, odds ratio; CI, confidence interval. * for *p* < 0.05, ** for *p* < 0.01.

The IVW method was predominant in our MR analysis. Notably, the levels of Omega-3 fatty acids (OR = 1.0014, 95% CI 1.0004–1.0024, *p* = 0.004), the ratio of DHA/totalFA (DHA/totalFA; OR = 1.0015, 95% CI 1.0002–1.0028, *p* = 0.023), and the ratio of Omega-3/totalFA (Omega-3/totalFA; OR = 1.0013, 95% CI 1.0003–1.0022, *p* = 0.010) were identified as biomarkers associated with an increased risk of colon polyps. Conversely, the ratio of Omega-6/Omega-3 (OR = 0.9986, 95% CI 0.9976–0.9995, *p* = 0.003) and the ratio of LA/totalFA (LA/totalFA; OR = 0.9981, 95% CI 0.9962–0.9999, *p* = 0.044) were negatively associated with susceptibility to colon polyps (Figure 2).

The MR-Egger, weighted median, and weighted mode analyses corroborated the trends observed with the IVW method, with the exception of the simple mode approach (as depicted in Figure 2).

In the case of other fatty acids (FAs), the IVW estimation did not reveal any significant causal associations with colon polyps. This lack of significant association was consistently supported by the results of other MR methods.

Upon conducting validation analyses with an alternative set of IVs, the causal relationships observed for Omega-3 fatty acid levels, the ratio of Omega-3/totalFA, the ratio of DHA/totalFA, the ratio of Omega-6/Omega-3, and the ratio of LA/totalFA with respect to colon polyps were found to be stable (Figure S1).

### 3.2. Characteristics of Selected SNPs

In this study, we selected SNPs as IVs for the MR analysis. A summary of information on these genetic instruments related to the levels of Omega-3 fatty acids, the ratio of Omega-3/totalFA, the ratio of DHA/totalFA, the ratio of Omega-6/Omega-3, and the ratio of LA/totalFA is presented in Supplementary Table S3. Palindromic variants and SNPs with high LD were excluded from the analysis. Cochrane’s Q statistic was utilized to assess heterogeneity among SNP-specific causal estimates for the five exposures, indicating no significant heterogeneity in the MR analysis. Each SNP demonstrated F-statistics above 10, with values ranging from 30.1 to 8976.9, which suggests that the potential for weak instrument bias is negligible.

### 3.3. Sensitivity and Heterogeneity Analysis

Heterogeneity in MR analyses can stem from variations in platforms, conditions, cohorts, and SNPs, which may influence causal effect estimations. We employed both the IVW method and MR-Egger regression to evaluate heterogeneity in our study. Furthermore, we applied Cochran’s Q test to evaluate the level of heterogeneity, considering a *p*-value below 0.05 as indicative of significant heterogeneity.

Cochran’s MR-Egger regression and IVW analyses showed no heterogeneity for SNPs linked to Omega-3 levels and colon polyps (*p* = 0.374 and 0.241, respectively). Similarly, no heterogeneity was observed between the ratios of Omega-3/totalFA, DHA/totalFA, Omega-6/Omega-3, LA/totalFA, and colon polyps (MR-Egger *p* = 0.150, IVW *p* = 0.125). These findings are detailed in Table 2.

The MR-Egger regression intercept analysis revealed no indication of significant horizontal pleiotropy in our MR study, with all *p*-values exceeding 0.05. This absence of significant pleiotropy suggests that our results are not only reliable but also highly robust. The details of these analyses are presented in Table 2.

**Figure 3:**
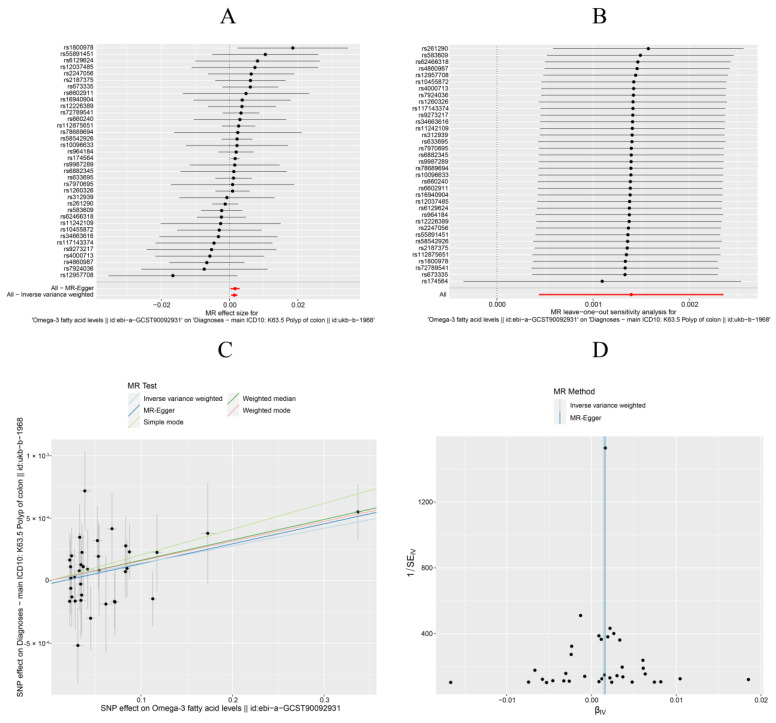
Mendelian randomization (MR) analysis of the causality of Omega-3 with the risk of colon polyps. (**A**) Forest plots of selected SNPs. (**B**) Sensitivity analysis. (**C**) Scatter plot. (**D**) Funnel plot.

Forest plots displayed the outcome estimates for individual SNPs. Upon examination, no single SNP was found to exert a substantial impact on the assessment of causal associations, which further substantiates the stability of our results (Figure 3A). Consequently, we deem the results of the IVW analysis to be reliable.

In our sensitivity analysis, a leave-one-out approach was implemented, as depicted in Figure 3B. Upon sequentially omitting each SNP, no significant changes were observed in the results. Consequently, the sensitivity analysis confirmed the reliability of the results from the MR analysis, showing no influence from heterogeneity or pleiotropy.

Scatter plots illustrated the effect size for each MR method, depicting the link between fatty acid exposure and the outcome. The scatter plot analysis indicates that Omega-3 was potentially associated with an increased risk of colon polyps (Figure 3C). Conversely, a higher ratio of Omega-6 to Omega-3 may be a risk factor for colon polyps, as depicted in the Supplementary Materials.

The funnel plot’s symmetrical arrangement of point estimates with a single SNP as an IV suggested minimal significant bias on the causal estimate, as illustrated in Figure 3D.

### 3.4. MR Study on Omega-3 Fatty Acids and Polyps in Other Body Systems

In our study, we aimed to explore the potential impact of Omega-3 fatty acids on the development of polyps across various body systems. Utilizing SNPs that affect Omega-3 levels as IVs, we gathered data from open GWAS datasets on multiple systems with diagnosed polyps, including nasal, bladder, gastroduodenal, rectal, female reproductive tract, uterine, and cervical polyps, as outcome variables (Table S2). To analyze the causal relationship, we employed five statistical methods, namely IVW, MR-Egger, weighted median, and weighted mode approaches. Additionally, we conducted an analysis to assess variable pleiotropy and heterogeneity.

**Figure 4:**
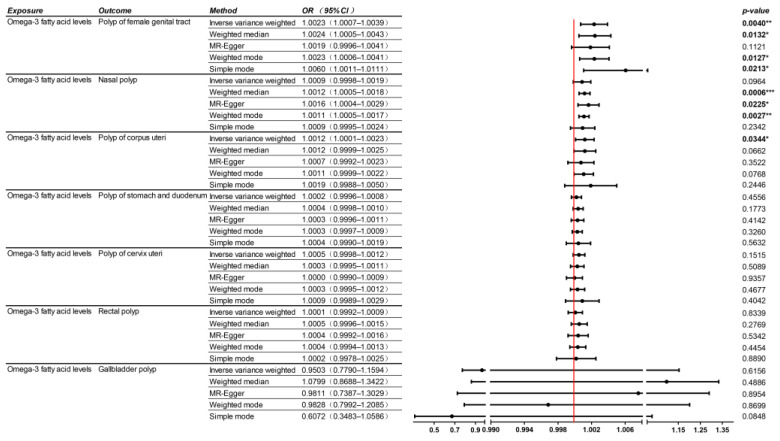
Mendelian randomization (MR) analysis of the causal relationships between Omega-3 and polyps in other body systems. OR, odds ratio; CI, confidence interval. * for *p* < 0.05, ** for *p* < 0.01, and *** for *p* < 0.001.

The IVW analysis indicated that higher levels of Omega-3 fatty acids are linked to a greater risk of polyps in the female reproductive tract, with no evidence of pleiotropy or heterogeneity within this analysis. Although the IVW analysis did not disclose a significant statistical association regarding the risk of nasal polyps, the MR-Egger, weighted median, and weighted mode models each showed significant correlations. Notably, significant heterogeneity was detected in the analyses assessing variable pleiotropy and heterogeneity. For comprehensive results, please refer to Figure 4.

## 4. Discussion

In this study, both the discovery and validation phases suggested a link between higher serum levels of Omega-3 fatty acids and an increased risk of colon polyps, whereas the sensitivity analysis showed that horizontal pleiotropy did not notably affect the findings of this study.

Omega-3 fatty acids, a class of PUFAs, are essential for human health as they cannot be synthesized de novo by the human body [18]. Consequently, dietary intake is the primary means of acquiring these nutrients. Omega-6 and Omega-3 fatty acids are metabolically and functionally distinct and are not interconvertible. They often serve opposing physiological roles, underscoring the importance of maintaining a balanced dietary intake of both [19].

Omega-3 fatty acids have been shown to have a protective effect against the development of psychiatric disorders, especially in high-risk adolescent groups [20]. It has been shown Omega-3 fatty acids contribute to the reduction of triglyceride levels in the blood, thereby potentially diminishing the risk of atherosclerosis and, consequently, the incidence of cardiovascular diseases [21]. Moreover, research has demonstrated that treating pregnancy-associated hypertriglyceridemia with a low-fat, fish-rich diet may reduce the risk of complications, such as altered utero-umbilical Doppler parameters and increased systemic arterial stiffness, potentially leading to elevated uteroplacental artery resistance [22]. Furthermore, Omega-3 fatty acids are recognized for their ability to suppress the production of inflammatory mediators, such as prostaglandins and leukotrienes, which can mitigate inflammatory responses [23].

The mechanisms of action of Omega-3 fatty acids encompass their ability to alter the fatty acid composition of cell membranes, which enhances membrane fluidity and, in turn, affects the functionality of membrane proteins [24]. This modulation can influence intracellular signaling pathways, including the reduction of inflammatory mediator production by limiting the availability of arachidonic acid [25]. Moreover, Omega-3 fatty acids exert antioxidant effects, mitigating oxidative stress and protecting cells from damage [26].

Interestingly, our research has revealed that Omega-3 fatty acids may play a detrimental role in the development of colon polyps and polyps in the female reproductive tract. Notably, glycerolipid metabolism has been identified as an aberrant metabolic pathway. Elevated levels of lipids and PUFAs, coupled with reduced levels of glycerol, suggest that glycerolipid metabolism is abnormal in colorectal polyps [12].

Omega-3 fatty acids, particularly the long-chain varieties eicosapentaenoic acid (EPA) and DHA, are prone to oxidation within the body due to their high content of double bonds [27]. This oxidative process can generate free radicals, which are highly reactive and capable of causing damage to cellular membranes, proteins, and DNA, potentially leading to cellular dysfunction and tissue injury [28]. An appropriate intake of Omega-3 fatty acids can provide health benefits without inducing significant oxidative damage as the body’s antioxidant systems are designed to handle a moderate level of oxidative stress. However, excessive intake of Omega-3 fatty acids, particularly in the absence of sufficient antioxidants, may lead to increased oxidative damage [29]. Actually, inflammation and oxidative damage are closely associated with the development of polyps. In recent years, research has highlighted the potential of natural compounds, such as curcumin and anthocyanins, to inhibit polyp formation and slow their progression to cancer, which is attributed to their antioxidant and anti-inflammatory properties [30,31].

MR studies uniquely infer causality by controlling for conventional confounders and eliminating reverse causality. Leveraging the largest genetic association dataset for fatty acids, comprising GWAS data from a discovery cohort of 135,006 samples and a corresponding validation set with 114,999 samples, this study explored the causal link between PUFAs and colon polyp risk using MR methods. The SNP data, sourced from a European cohort, aimed to reduce biases arising from population differences. The results suggest that higher serum Omega-3 levels may causally increase colon polyp risk. Additionally, a higher Omega-6/Omega-3 ratio might be associated with increased risk, whereas no causal link was found for Omega-6 alone. The sensitivity analysis confirmed no significant impact from heterogeneity or pleiotropy. Although the IVW method may offer greater efficiency in identifying causal effects compared to weighted median and MR-Egger analyses, its strong assumptions could result in a higher type I error rate and bias in causal effect estimation [32,33]. Despite this, our findings utilizing the MR-Egger, weighted median, and weighted mode methods were less susceptible to issues of horizontal multiplicity, yielding results that were comparable to those of the IVW estimates.

## 5. Strengths and Limitations

Our study also has its limitations. The data predominantly reflect individuals of European descent, which, though beneficial for reducing racial confounding, may restrict the generalizability of our findings to other ethnic groups. Another aspect that requires careful consideration is the population heterogeneity within European cohorts. For instance, in terms of genetic fluidity, the Mediterranean demographic has likely experienced a more intricate mosaic of gene flow dynamics compared to their Northern European counterparts. This complexity is characterized by significant interactions with regions as diverse as the Middle East and North Africa, which have shaped a unique genetic heritage. Indeed, variability in the response to daily lipid-lowering medications among different populations can also influence the levels of fatty acids. It has been shown that Rosuvastatin calcium, a lipid-lowering medication, exhibits significant pharmacokinetic differences among racial populations. In Asian patients, the drug’s exposure has been observed to be twice as high as in Caucasian individuals. This suggests that the causal relationship between polyunsaturated fatty acids (PUFAs) and colon polyps may be more complex in other ethnic groups.

Furthermore, the dataset for colon polyps is comparatively limited, preventing comprehensive multi-database validation. The potential for sample overlap, due to the shared European origin of both exposure and outcome data, also exists. Accurately determining the extent of this overlap is challenging. Considering the potential variations in the prevalence of colorectal polyps between men and women as reported in studies [34,35], we cannot dismiss the possibility that gender may be a confounding factor. Another point worth noting is that, although the *p*-value for the causal relationship between PUFAs and colon polyps in the MR analysis was significantly meaningful, the OR value was close to 1. Therefore, future analyses with larger samples are needed. Consequently, further exploration through randomized controlled trials (RCTs) is essential. RCTs provide a higher level of certainty and stringent controls, which are necessary to validate the inferred causality and establish a cause–effect relationship.

## 6. Conclusions

In conclusion, our MR study suggests a possible adverse effect of PUFAs, especially Omega-3 fatty acids, on colon polyp development, with higher Omega-3 levels linked to a higher risk. Although Omega-3 fatty acids have been widely recognized for their preventative effects against numerous diseases, it is imperative that their potential adverse effects are not overlooked.

## Figures and Tables

**Table 1:** Summary of GWAS datasets of discovery exposures and outcome.

Exposure	GWAS ID	Year	Consortium	Sample Size	Number of SNPs
MUFA	ebi-a-GCST90092928	2022	NA	115,006	11,590,399
Omega-3	ebi-a-GCST90092931	2022	NA	115,006	11,590,399
Omega-6	ebi-a-GCST90092933	2022	NA	115,006	11,590,399
PUFA	ebi-a-GCST90092939	2022	NA	115,006	11,590,399
DHA/totalFA	ebi-a-GCST90092817	2022	NA	115,006	11,590,399
LA/totalFA	ebi-a-GCST90092881	2022	NA	115,006	11,590,399
MUFA/totalFA	ebi-a-GCST90092929	2022	NA	115,006	11,590,399
Omega-3/totalFA	ebi-a-GCST90092932	2022	NA	115,006	11,590,399
Omega-6/Omega-3	ebi-a-GCST90092934	2022	NA	115,006	11,590,399
Omega-6/totalFA	ebi-a-GCST90092935	2022	NA	115,006	11,590,399
PUFA/MUFA	ebi-a-GCST90092940	2022	NA	115006	11,590,399
PUFA/totalFA	ebi-a-GCST90092941	2022	NA	115,006	11,590,399
SFA/totalFA	ebi-a-GCST90092981	2022	NA	115,006	11,590,399
SFA	ebi-a-GCST90092980	2022	NA	115,006	11,590,399
totalFA	ebi-a-GCST90092987	2022	NA	115,006	11,590,399
**Outcome**	**GWAS ID**	**Year**	**Consortium**	**Sample Size**	**Number of SNPs**
Polyp of colon	ukb-b-1968	2018	MRC-IEU	463,010	9,851,867

**Table 2:** Pleiotropy test and heterogeneity test of the exposures from MR.

	Pleiotropy Test			Heterogeneity Test					
	MR-Egger			MR-Egger			IVW		
	Intercept	SE	*p*-Value	*Q*	Q (df)	*p*-Value	*Q*	Q (df)	*p*-Value
Omega-3	4.81 × 10^−5^	5.85 × 10^−5^	0.417	24.20	34	0.893	24.39	35	0.910
DHA/totalFA	−5.68 × 10^−5^	7.32 × 10^−5^	0.447	13.74	21	0.880	14.34	22	0.889
LA/totalFA	1.03 × 10^−4^	7.56 × 10^−5^	0.185	33.74	28	0.210	35.96	29	0.175
Omega-3/totalFA	−7.45 × 10^−5^	6.01 × 10^−5^	0.227	20.27	26	0.778	21.81	27	0.747
Omega-6/Omega-3	2.28 × 10^−5^	6.72 × 10^−5^	0.738	11.39	23	0.979	11.51	24	0.985

## Data Availability

Data are contained within the article and Supplementary Materials.

## Supplementary Materials

The following supporting information can be downloaded at: https://www.mdpi.com/article/10.3390/nu16132033/s1, Figure S1: Mendelian randomization (MR) analysis showing the causal relationship between different types of FAs and colon polyp outcomes in the validation cohorts; Table S1: Summary of GWAS datasets of exposure and outcome in the validate cohorts; Table S2: Summary of GWAS datasets of Omega-3 fatty acids and polyps in other body systems; Table S3 Characteristics of selected SNPs.
