# Supplementary material for: Causal Relationships between Polyunsaturated Fatty Acids and Colon Polyps: A Two-Sample Mendelian Randomization Study
Source: Nutrients. 2024 Jun 27;16(13):2033. doi: 10.3390/nu16132033 (PMC11243184; doi:10.3390/nu16132033)

Exposure	***Outcome***	***Method***	***OR (95%CI)***	***P-value***
MUFA	polyp of colon	Inverse variance weighted	1.0007(0.9993-1.0020)	0.3211
		Weighted median	1.0009(0.9990-1.0028)	0.3484
		MR Egger	1.0001(0.9978-1.0024)	0.9322
		Weighted mode	1.0006(0.9988-1.0023)	0.5302
		Simple mode	1.0009(0.9977-1.0042)	0.5821
Omega-3	polyp of colon	Inverse variance weighted	1.0014(1.0004-1.0023)	**0.0041****
		Weighted median	1.0016(1.0004-1.0029)	**0.0094****
		MR Egger	1.0016(1.0003-1.0030)	**0.0217***
		Weighted mode	1.0016(1.0004-1.0028)	**0.0132***
		Simple mode	1.0022(0.9994-1.0049)	0.1263
Omega-6	polyp of colon	Inverse variance weighted	1.0009(0.9994-1.0024)	0.2259
		Weighted median	1.0013(0.9993-1.0033)	0.2101
		MR Egger	1.0004(0.9973-1.0036)	0.7862
		Weighted mode	1.0010(0.9987-1.0033)	0.3921
		Simple mode	1.0024(0.9990-1.0058)	0.1681
PUFA	polyp of colon	Inverse variance weighted	1.0012(0.9999-1.0025)	0.0770
		Weighted median	1.0013(0.9995-1.0031)	0.1600
		MR Egger	1.0017(0.9990-1.0044)	0.2156
		Weighted mode	1.0012(0.9990-1.0033)	0.2986
		Simple mode	1.0020(0.9988-1.0052)	0.2337
DHA/totalFA	polyp of colon	Inverse variance weighted	1.0015(1.0002-1.0028)	**0.0251***
		Weighted median	1.0019(1.0003-1.0034)	**0.0166***
		MR Egger	1.0020(1.0002-1.0038)	**0.0458***
		Weighted mode	1.0020(1.0004-1.0036)	**0.0204***
		Simple mode	0.9980(0.9936-1.0025)	0.3917
LA/totalFA	polyp of colon	Inverse variance weighted	0.9980(0.9962-0.9999)	**0.0382***
		Weighted median	0.9973(0.9951-0.9994)	**0.0121***
		MR Egger	0.9967(0.9938-0.9996)	**0.0335***
		Weighted mode	0.9974(0.9952-0.9995)	**0.0217***
		Simple mode	0.9963(0.9905-1.0021)	0.2188
MUFA/totalFA	polyp of colon	Inverse variance weighted	0.9998(0.9985-1.0011)	0.7988
		Weighted median	0.9989(0.9970-1.0007)	0.2335
		MR Egger	0.9984(0.9965-1.0003)	0.1123
		Weighted mode	0.9993(0.9978-1.0009)	0.3987
		Simple mode	1.0009(0.9980-1.0038)	0.5481
Omega-3/totalFA	polyp of colon	Inverse variance weighted	1.0013(1.0003-1.0023)	**0.0082****
		Weighted median	1.0014(1.0004-1.0025)	**0.0095****
		MR Egger	1.0016(1.0003-1.0028)	**0.018***
		Weighted mode	1.0015(1.0005-1.0026)	**0.0092****
		Simple mode	1.0031(0.9991-1.0071)	0.1375
Omega-6/Omega-3	polyp of colon	Inverse variance weighted	0.9986(0.9976-0.9996)	**0.0046****
		Weighted median	0.9985(0.9974-0.9995)	**0.0053****
		MR Egger	0.9984(0.9971-0.9997)	**0.0218***
		Weighted mode	0.9984(0.9973-0.9995)	**0.0095****
		Simple mode	0.9969(0.9934-1.0004)	0.0957
Omega-6/totalFA	polyp of colon	Inverse variance weighted	0.9995(0.9979-1.0011)	0.5405
		Weighted median	0.9993(0.9971-1.0015)	0.5364
		MR Egger	0.9998(0.9969-1.0026)	0.8816
		Weighted mode	0.9997(0.9973-1.0022)	0.8235
		Simple mode	1.0001(0.9964-1.0038)	0.9597
PUFA/MUFA	polyp of colon	Inverse variance weighted	1.0003(0.9991-1.0016)	0.6016
		Weighted median	1.0008(0.9986-1.0029)	0.4768
		MR Egger	1.0016(0.9996-1.0037)	0.1280
		Weighted mode	1.0007(0.9988-1.0025)	0.4969
		Simple mode	0.9998(0.9965-1.0032)	0.9297
PUFA/totalFA	polyp of colon	Inverse variance weighted	1.0004(0.9988-1.0020)	0.6335
		Weighted median	0.9998(0.9973-1.0023)	0.8729
		MR Egger	1.0019(0.9990-1.0048)	0.2079
		Weighted mode	0.9992(0.9963-1.0021)	0.5889
		Simple mode	0.9983(0.9943-1.0024)	0.4268
SFA/totalFA	polyp of colon	Inverse variance weighted	1.0005(0.9974-1.0036)	0.7498
		Weighted median	0.9986(0.9948-1.0025)	0.4804
		MR Egger	0.9988(0.9872-1.0105)	0.8410
		Weighted mode	0.9983(0.9929-1.0037)	0.5341
		Simple mode	0.9984(0.9925-1.0043)	0.5936
SFA	polyp of colon	Inverse variance weighted	1.0016(1.0001-1.0030)	**0.0309***
		Weighted median	1.0008(0.9987-1.0030)	0.4532
		MR Egger	0.9990(0.9964-1.0016)	0.4520
		Weighted mode	1.0005(0.9983-1.0027)	0.6625
		Simple mode	1.0012(0.9979-1.0044)	0.4845
totalFA	polyp of colon	Inverse variance weighted	1.0011(0.9998-1.0024)	0.0868
		Weighted median	1.0011(0.9993-1.0030)	0.2294
		MR Egger	0.9996(0.9973-1.0019)	0.7530
		Weighted mode	1.0007(0.9987-1.0027)	0.4805
		Simple mode	1.0016(0.9985-1.0046)	0.3263

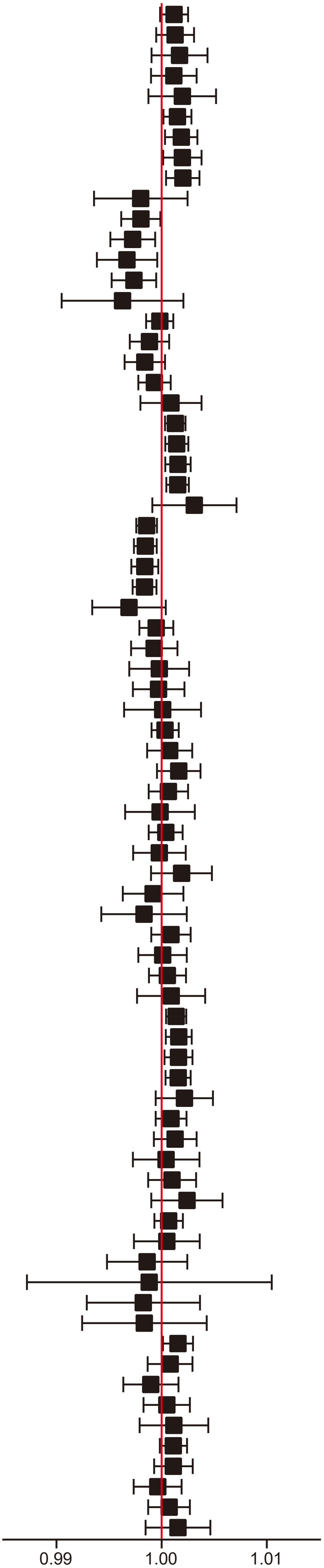

Supplement: Supplementary file 1 [file nutrients-16-02033-s001.zip › Figure S1.pdf]
